# Crystal structure, Hirshfeld surface analysis and inter­action energy and DFT studies of 1-(1,3-benzo­thia­zol-2-yl)-3-(2-hy­droxy­eth­yl)imidazolidin-2-one

**DOI:** 10.1107/S2056989020001723

**Published:** 2020-02-14

**Authors:** Mohamed Srhir, Nada Kheira Sebbar, Tuncer Hökelek, Ahmed Moussaif, Joel T. Mague, Noureddine Hamou Ahabchane, El Mokhtar Essassi

**Affiliations:** a Laboratoire de Chimie Organique Hétérocyclique URAC 21, Pôle de Compétence Pharmacochimie, Av. Ibn Battouta, BP 1014, Faculté des Sciences, Université Mohammed V, Rabat, Morocco; b Faculté des Sciences Appliquées Ait Melloul, Université Ibn Zohr, Agadir, Morocco; c Department of Physics, Hacettepe University, 06800 Beytepe, Ankara, Turkey; d Department of Chemistry, Tulane University, New Orleans, LA 70118, USA

**Keywords:** crystal structure, benzo­thia­zine, hydrogen bond, triazole, π-stacking, Hirshfeld surface

## Abstract

In the title mol­ecule, C_12_H_13_N_3_O_2_S, the benzo­thia­zine moiety is slightly non-planar, with the imidazolidine portion twisted only a few degrees out of the mean plane of the former. In the crystal, a layer structure parallel to the *bc* plane is formed by a combination of O—H_Hydethy_⋯N_Thz_ hydrogen bonds and weak C—H_Imdz_⋯O_Imdz_ and C—H_Bnz_⋯O_Imdz_ (Hydethy = hy­droxy­ethyl, Thz = thia­zole, Imdz = imidazolidine and Bnz = benzene) inter­actions, together with C—H_Imdz_⋯π(ring) and head-to-tail slipped π-stacking [centroid-to-centroid distances = 3.6507 (7) and 3.6866 (7) Å] inter­actions between thia­zole rings. The Hirshfeld surface analysis of the crystal structure indicates that the most important contributions for the crystal packing are from H⋯H (47.0%), H⋯O/O⋯H (16.9%), H⋯C/C⋯H (8.0%) and H⋯S/S⋯H (7.6%) inter­actions. Hydrogen bonding and van der Waals inter­actions are the dominant inter­actions in the crystal packing. Computational chemistry indicates that in the crystal, C—H⋯N and C—H⋯O hydrogen-bond energies are 68.5 (for O—H_Hydethy_⋯N_Thz_), 60.1 (for C—H_Bnz_⋯O_Imdz_) and 41.8 kJ mol^−1^ (for C—H_Imdz_⋯O_Imdz_). Density functional theory (DFT) optimized structures at the B3LYP/6–311 G(d,p) level are compared with the experimentally determined mol­ecular structure in the solid state.

## Abstract 2

The title mol­ecule is only a few degrees out of planarity except for the 2-hy­droxy­ethyl substituent. In the crystal, O—H⋯N hydrogen bonds form stepped chains along the *c*-axis direction, which are formed into layers parallel to the *bc* plane by weak C—H⋯O and C—H⋯π (ring) inter­actions. Completion of the overall layer structure occurs through weak C—H⋯O, C—H⋯π (ring) and head-to-tail slipped π-stacking inter­actions.

## Chemical context   

Compounds containing the benzo­thia­zole backbone have been studied extensively in both academic and industrial laboratories (Mekhzoum *et al.*, 2016 ▸, 2019 ▸; Chakib *et al.*, 2010*a* ▸,*b* ▸, 2019 ▸). These mol­ecules exhibit a wide range of biological applications including as anti-tumor agents (Bénéteau *et al.*, 1999 ▸; Ćaleta *et al.*, 2004 ▸), anti­microbial agents (Shastry *et al.*, 2003 ▸; Latrofa *et al.*, 2005 ▸, Singh *et al.*, 2013 ▸), analgesics (Kaur *et al.*, 2010 ▸), anti-inflammatory agents (Oketani *et al.*, 2001 ▸), anti-HIV agents (Nagarajan *et al.*, 2003 ▸; Pitta *et al.*, 2013 ▸), anti-leishmanial agents (Delmas *et al.*, 2004 ▸), anti-cancer agents (Yang *et al.*, 2003 ▸; Huang *et al.*, 2006 ▸; Kok *et al.*, 2008 ▸), anti-hypertensive agents (Saggu *et al.*, 2002 ▸), anti­oxidants, (Ayhan-Kilcigil *et al.*, 2004 ▸) and anti-viral agents (Tewari *et al.*, 2006 ▸). The imidazolinone moiety is an important scaffold possessing a spectrum of pharmacological actions, which include anti-convulsant, anti-parkinsonism and mono­amino-oxidase inhibitory activities (Hari Narayana Moorthy *et al.*, 2012 ▸; Desai *et al.*, 2009 ▸). Furthermore, imidazolo­nes are anti-bacterial, anti-fungal, anti-viral, anti-cancer and CNS-depressant agents (Naithani *et al.*, 1989 ▸; Harfenist *et al.*, 1978 ▸). We have previously shown that bis­(2-chloro­eth­yl)amine hydro­chloride is an inter­esting precursor of several heterocyclic compounds containing the oxazolidinone moiety (Sebbar *et al.*, 2016 ▸, 2018 ▸; Ellouz *et al.*, 2017 ▸; Hni *et al.*, 2019 ▸). In a continuation of our research using bis­(2-chloro­eth­yl)amine hydro­chloride as an inter­mediate in the synthesis of new heterocyclic systems, we have studied the condensation of 2-amino­benzo­thia­zole with bis­(2-chloro­eth­yl)amine hydro­chloride in the presence of tetra-*n*-butyl­ammonium bromide as catalyst and potassium carbonate as base. A plausible mechanism for the formationof the product, 1-(1,3-benzo­thia­zol-2-yl)-3-(2-hy­droxy­eth­yl)imid­azolidin-2-one (**I**), is given in the reaction scheme.
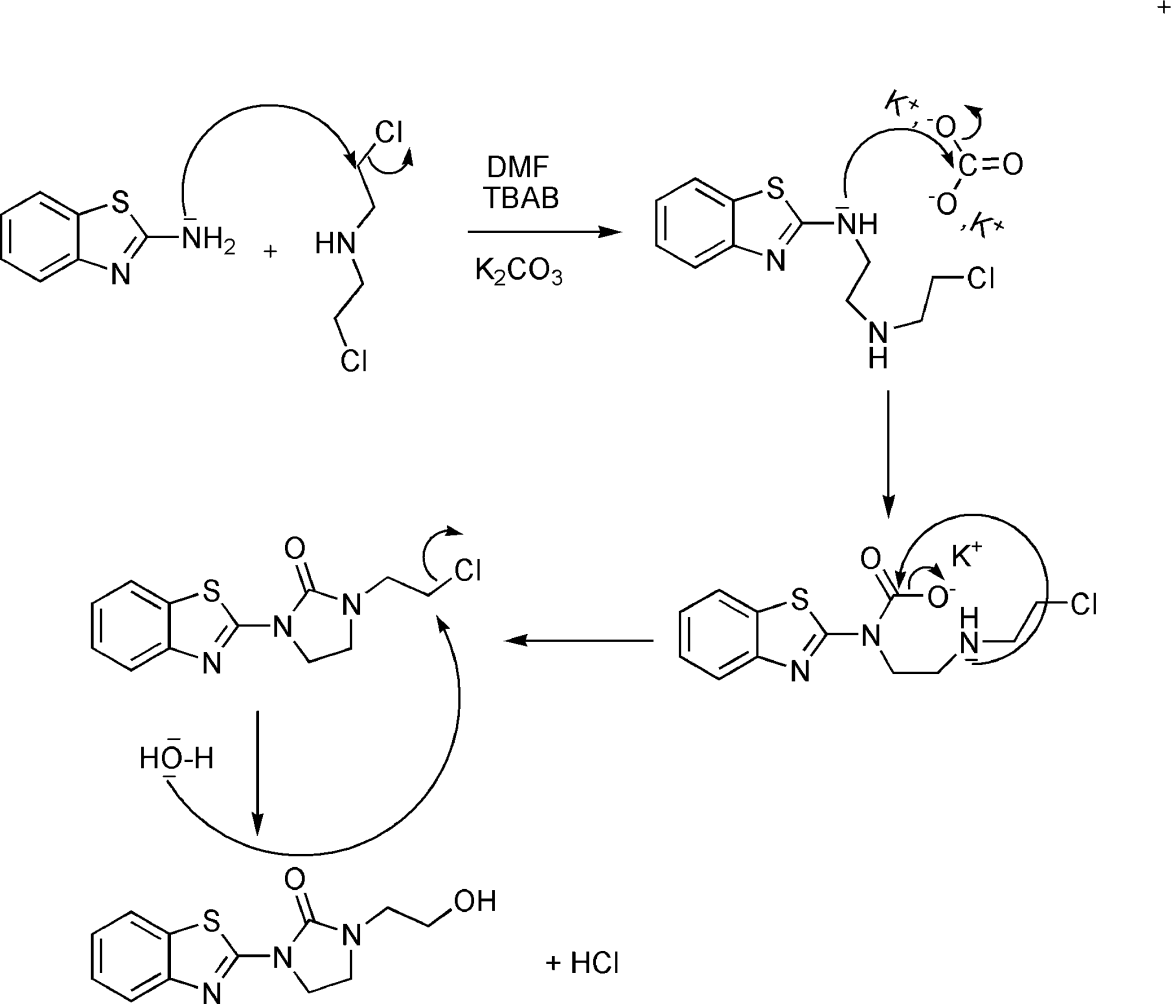


The title compound was obtained for the first time and characterized by single crystal X-ray diffraction techniques as well as by Hirshfeld surface analysis. The results of the calculations by density functional theory (DFT), carried out at the B3LYP/6-311G (d,p) level, are compared with the experimentally determined mol­ecular structure in the solid state.
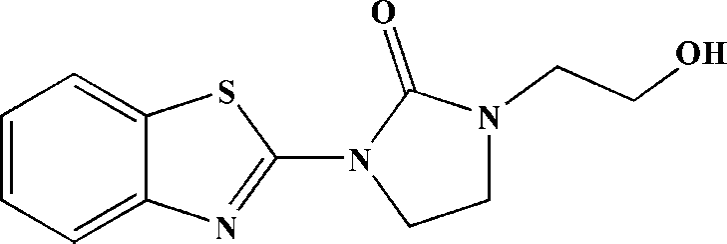


## Structural commentary   

**Figure 1:**
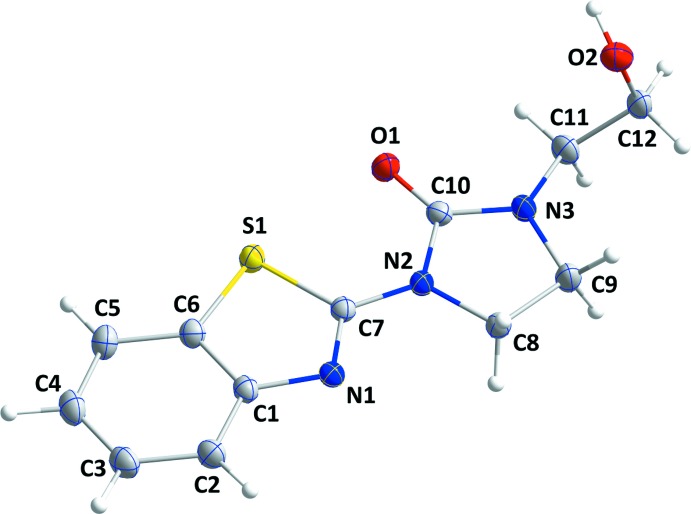
The mol­ecular structure of the title compound with the atom-numbering scheme. Displacement ellipsoids are drawn at the 50% probability level.

In the title mol­ecule (**I**) (Fig. 1 ▸), the benzo­thia­zole unit is slightly non-planar, as indicated by the dihedral angle of 1.52 (4)° between the mean planes of the component rings, [*A* (C1–C6) and *B* (S1/N1/C1/C6/C7)]. A puckering analysis of the conformation of the imidazolidine ring *C* (N2/N3/C8-C10) gave the parameters *Q*(2) = 0.0767 (14) Å and φ(2) = 66.5 (10)°. The conformation is described as an ‘envelope on C9′. This ring is almost coplanar with the thia­zole ring *B* with a dihedral angle of 3.61 (4)° between their mean planes.

## Supra­molecular features   

**Figure 2:**
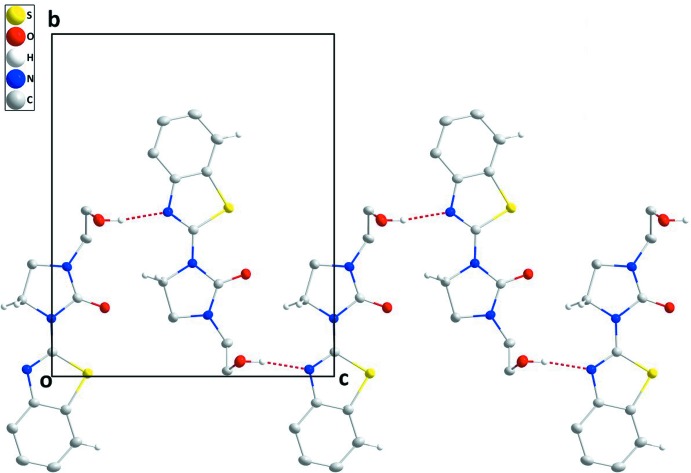
A portion of the O—H_Hydethy_⋯N_Thz_ (Hydethy = hy­droxy­ethyl and Thz = thia­zole) (red dashed lines) hydrogen bonded chain in **I** viewed along the *a* axis.

**Figure 3:**
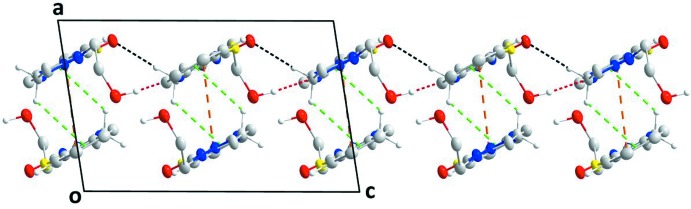
Portions of two chains, viewed along the *b* axis, showing the inter­actions between them. O—H_Hydethy_⋯N_Thz_ hydrogen bonds are shown by red dashed lines while the weak C—H_Imdz_⋯O_Imdz_ and C—H_Bnz_⋯O_Imdz_ (Hydethy = hy­droxy­ethyl, Thz = thia­zole, Imdz = imidazolidine and Bnz = benzene) inter­actions are shown by black dashed lines. The weak C—H_Imdz_⋯π(ring) and the head-to-tail slipped π-stacking inter­actions are shown, respectively, by green and orange dashed lines.

In the crystal, O—H_Hydethy_⋯N_Thz_ (Hydethy = hy­droxy­ethyl and Thz = thia­zole) hydrogen bonds (Table 1 ▸) form stepped chains of mol­ecules extending along the *c*-axis direction (Fig. 2 ▸). These are connected into layers parallel to the *bc* plane by weak C—H_Imdz_⋯O_Imdz_ (Imdz = imidazolidine) and C—H_Imdz_⋯π(ring) inter­actions (Table 1 ▸). The layers are connected by weak C—H_Bnz_⋯O_Imdz_ (Bnz = benzene) inter­actions. Both the layer formation and stacking are also assisted by head-to-tail slipped π-stacking inter­actions (Figs. 3 ▸ and 4 ▸) along the *a*-axis direction between thia­zole rings [*Cg*2⋯*Cg*2^i^ and *Cg*2⋯*Cg*2^ii^ = 3.6507 (7) and 3.6866 (7) Å, respectively; symmetry codes: (i) −*x* + 1, −*y* + 1, −*z* + 1; (ii) −*x* + 2, −*y* + 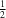, −*z* + 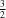, where *Cg*2 is the centroid of ring *B*].

## Hirshfeld surface analysis   

**Figure 5:**
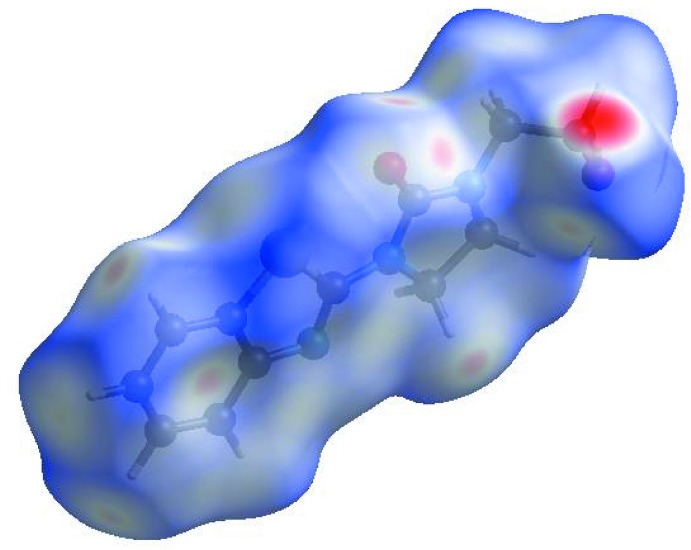
View of the three-dimensional Hirshfeld surface of **I** plotted over *d*_norm_ in the range −0.5793 to 1.2827 a.u.

**Figure 6:**
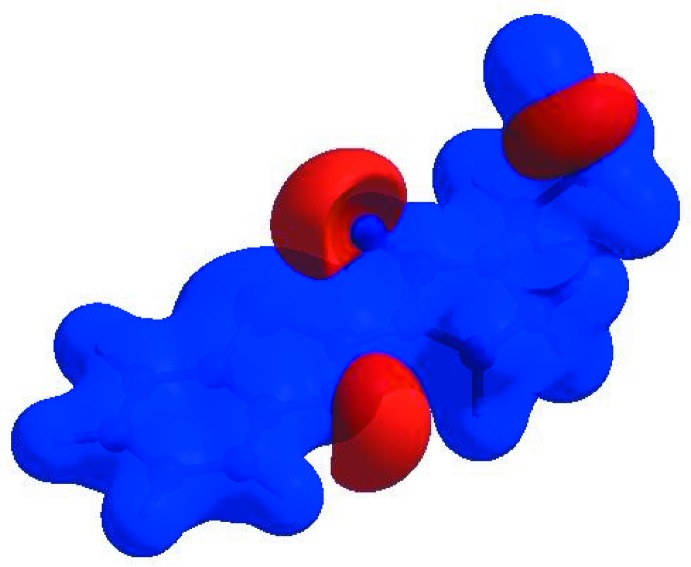
View of the three-dimensional Hirshfeld surface of **I** plotted over electrostatic potential energy in the range −0.0500 to 0.0500 a.u. using the STO-3 G basis set at the Hartree–Fock level of theory. Hydrogen-bond donors and acceptors are shown as blue and red regions around the atoms corresponding to positive and negative potentials, respectively.

**Figure 7:**
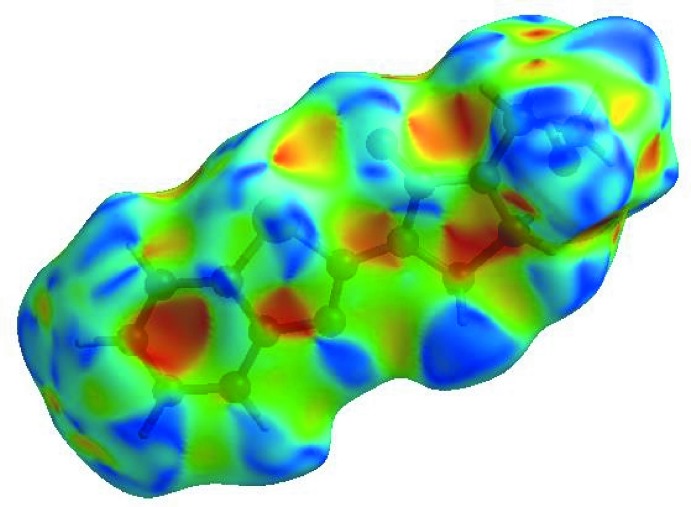
Hirshfeld surface of **I** plotted over shape-index.

In order to visualize the inter­molecular inter­actions in the crystal of **I**, a Hirshfeld surface (HS) analysis (Hirshfeld, 1977 ▸; Spackman & Jayatilaka, 2009 ▸) was carried out using *Crystal Explorer 17.5* (Turner *et al.*, 2017 ▸). In the HS plotted over *d*_norm_ (Fig. 5 ▸), the white surface indicates contacts with distances equal to the sum of van der Waals radii, and the red and blue colours indicate distances shorter (in close contact) or longer (distant contact) than the van der Waals radii, respectively (Venkatesan *et al.*, 2016 ▸). The bright-red spots appearing near O1 and hydrogen atoms H5, H2*A*, H8*B* indicate their roles as donors and/or acceptors; they also appear as blue and red regions corresponding to positive and negative potentials on the HS mapped over electrostatic potential (Spackman *et al.*, 2008 ▸; Jayatilaka *et al.*, 2005 ▸) shown in Fig. 6 ▸. The blue regions indicate positive electrostatic potential (hydrogen-bond donors), while the red regions indicate negative electrostatic potential (hydrogen-bond acceptors). The shape-index of the HS is a tool to visualize π–π stacking by the presence of adjacent red and blue triangles; if there are no adjacent red and/or blue triangles, then there are no π–π inter­actions. Fig. 7 ▸ clearly suggests that there are π–π inter­actions in **I**.

**Figure 8:**
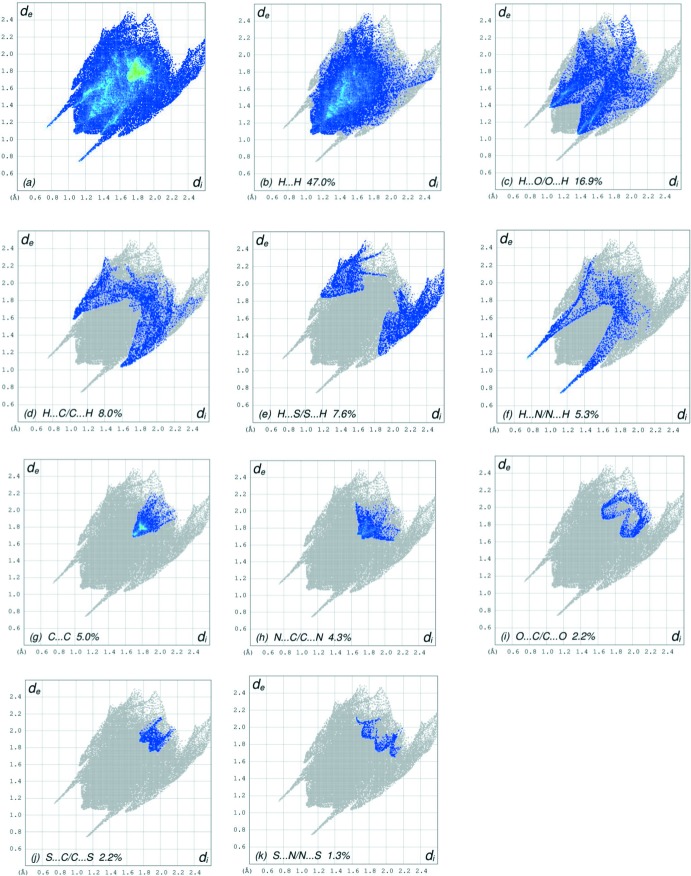
The full two-dimensional fingerprint plots for **I**, showing (*a*) all inter­actions, and those delineated into (*b*) H⋯H, (*c*) H⋯O/O⋯H, (*d*) H ⋯ C/C⋯H, (*e*) H⋯S/S⋯H, (*f*) H⋯N/N⋯H, (*g*) C⋯C, (*h*) C ⋯ N/N⋯C, (i) O⋯C/C⋯O, (*j*) S⋯C/C⋯S and (*k*) S⋯N/N⋯S inter­actions. The *d*_i_ and *d*_e_ values are the closest inter­nal and external distances (in Å) from given points on the Hirshfeld surface.

The overall two-dimensional fingerprint plot, Fig. 8 ▸ *a*, and those delineated into H⋯H, H⋯O/O⋯H, H⋯C/C⋯H, H⋯S/S⋯H, H⋯N/N ⋯ H, C⋯C, N⋯C/C⋯N, O⋯C/C⋯O, S⋯C/C⋯S and S⋯N/N ⋯ S contacts (McKinnon *et al.*, 2007 ▸) are illustrated in Fig. 8 ▸ *b*–*k*, respectively, together with their relative contributions to the Hirshfeld surface. The most important inter­action (Table 2 ▸) is H⋯H, contributing 47.0% to the overall crystal packing, which is reflected in Fig. 8 ▸ *b* as widely scattered points of high density due to the large hydrogen content of the mol­ecule with the tip at *d*_e_ = *d*_i_ = 1.10 Å. The pair of wings in the fingerprint plot delineated into H⋯O/O⋯H contacts (Fig. 8 ▸ *c*, 16.9% contribution) has a symmetrical distribution of points with the edges at *d*_e_ + *d*_i_ = 2.40 Å. The presence of C—H⋯π inter­actions is indicated by the characteristic wings with a spikes with the tips at *d*_e_ + *d*_i_ = 2.63 Å in the fingerprint plot delineated into H⋯C/C⋯H contacts (Fig. 8 ▸ *d*, 8.0% contribution). The H⋯S/S⋯H contacts contribute 7.6% to the overall crystal packing and are seen in Fig. 8 ▸ *e* as widely scattered points with the tips at *d*_e_ + *d*_i_ = 3.03 Å. The pair of spikes in the fingerprint plot delineated into H⋯N/N⋯H contacts (Fig. 8 ▸ *f*, 5.3%) has a symmetrical distribution of points with the tips at *d*_e_ + *d*_i_ = 1.88 Å. The C⋯C contacts (5.0% contribution, Fig. 8 ▸ *g*) have an arrow-shaped distribution of points with the tip at *d*_e_ = *d*_i_ = 1.70 Å. The N⋯C/C⋯N inter­actions (4.3%, Fig. 8 ▸ *h*) give rise to tiny wings with the tips at *d*_e_ + *d*_i_ = 3.41 Å. The O⋯C/C⋯O contacts (2.2%, Fig. 8 ▸ *i*) give widely scattered points with the tips at *d*_e_ + *d*_i_ = 3.56 Å. Finally, the S⋯C/C⋯S and S⋯N/N⋯S inter­actions, contributing 2.2% and 1.3% to the overall crystal packing (Fig. 8 ▸ *j* and *k*) give rise to tiny wings with the tips at *d*_e_ + *d*_i_ = 3.63 Å and *d*_e_ + *d*_i_ = 3.63 Å, respectively.

**Figure 9:**
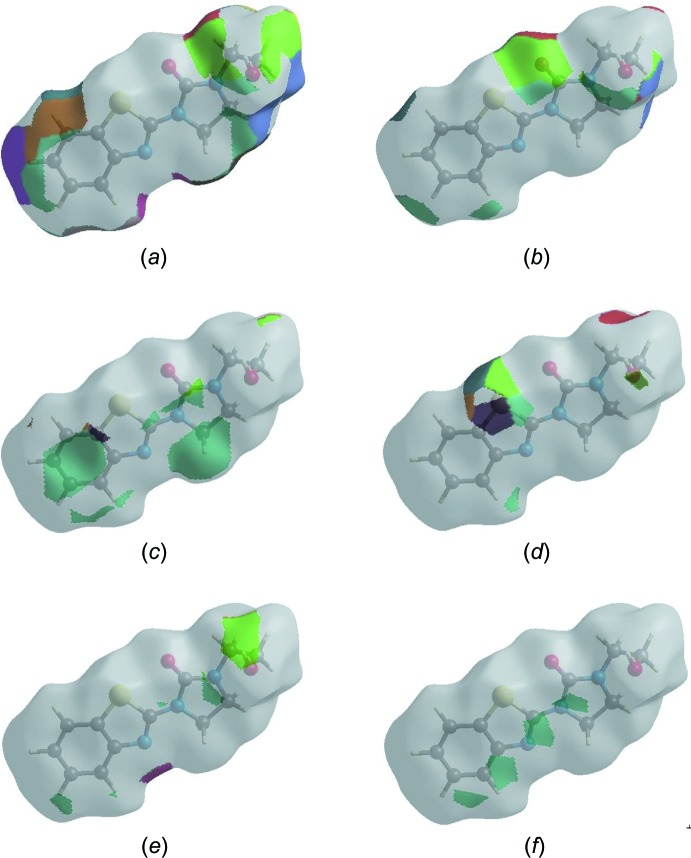
The Hirshfeld surface representations for **I** with the function *d*_norm_ plotted onto the surface for (*a*) H⋯H, (*b*) H⋯O/O⋯H, (*c*) H⋯C/C⋯ H, (*d*) H⋯S/S⋯H, (*e*) H⋯N/N⋯H and (*f*) C⋯C inter­actions.

The Hirshfeld surface representations with the function *d*_norm_ plotted onto the surface are shown for the H⋯H, H⋯O/O⋯H, H⋯C/C⋯H, H ⋯ S/S⋯H, H⋯N/N⋯H and C⋯C inter­actions in Fig. 9 ▸ *a-*-*f*, respectively.

The Hirshfeld surface analysis confirms the importance of H-atom contacts in establishing the packing. The large number of H⋯H, H⋯O/O⋯H and H⋯C/C⋯H inter­actions suggest that van der Waals inter­actions and hydrogen bonding play the major roles in the crystal packing (Hathwar *et al.*, 2015 ▸).

## Inter­action energy calculations   

The inter­molecular inter­action energies were calculated by the CE–B3LYP/6–311G(d,p) energy model available in *Crystal Explorer 17.5* (Turner *et al.*, 2017 ▸) using the cluster of mol­ecules generated by applying crystallographic symmetry operations within a radius of 3.8 Å of a central mol­ecule (Turner *et al.*, 2014 ▸). The total inter­molecular energy (*E*_tot_) is the sum of electrostatic (*E*_ele_), polarization (*E*_pol_), dispersion (*E*_dis_) and exchange-repulsion (*E*_rep_) energies (Turner *et al.*, 2015 ▸) with scale factors of 1.057, 0.740, 0.871 and 0.618, respectively (Mackenzie *et al.*, 2017 ▸). Hydrogen-bonding inter­action energies (in kJ mol^−1^) were calculated to be −67.2 (*E*_ele_), −18.0 (*E*_pol_), −35.4 (*E*_dis_), 75.7 (*E*_rep_) and −68.5 (*E*_tot_) for O2—H2*A*⋯N1, −21.5 (*E*_ele_), −6.1 (*E*_pol_), −82.0 (*E*_dis_), 62.3 (*E*_rep_) and −60.1 (*E*_tot_) for C5—H5⋯O1 and −1.2 (*E*_ele_), −6.3 (*E*_pol_), −73.7 (*E*_dis_), 45.7 (*E*_rep_) and −41.8 (*E*_tot_) for C8—H8*B*⋯O1.

## DFT calculations   

The main aim of these computations is to provide an inter­pretation of the experimental results. For this purpose, the structural parameters of equilibrium geometry for **I** in the gas phase have been computed using the B3LYP functional level of theory and the 6-31G (d,p) basis set (Becke, 1993 ▸) implemented in *GAUSSIAN-09* (Frisch *et al.*, 2009 ▸). The mol­ecule adopts a geometry very close to that obtained using DFT calculations (Table 3 ▸). The largest differences between the calculated and experimental values are observed for the S1—C6 (0.1 Å) and S1—C7 (0.08 Å) bond lengths and the C11—N3—C9 bond angle (1.6°). These disparities can be linked to the fact that these calculations relate to the isolated mol­ecule, whereas the experimental results correspond to inter­acting mol­ecules in the crystal lattice where intra and inter­molecular inter­actions with the neighboring mol­ecules are present.

## Database survey   

A search of the Cambridge Structural Database (CSD, Version 5.41 updated to December 2019; Groom *et al.*, 2016 ▸) with the search fragment **II** generated 24 hits of which 10 were metal complexes of benzo­thia­zole or its derivatives. Of the remaining mol­ecules, the four closest in composition and structure to **I** are **III** (KEQTAC; Olyaei *et al.*, 2006 ▸), **IV** (NOHJAX; Sahoo *et al.*, 2014 ▸), **V** (RUBPAG; Saczewski *et al.*, 2005 ▸) and **VI** (YUYTUH; Kozísek *et al.*, 1995 ▸). In all four, the benzo­thia­zole moiety is more nearly planar than in **I**, with the dihedral angle between the constituent planes being < 1° except for **VI** where it is 1.3°. In **I**, the dihedral angle between the planes defined by C7/N1/C1/C6/S1 and C7/N2/C8/C10 is 1.94 (4)° while the corresponding dihedral angle in the others vary from 13.64° in **III** to 0.61° in **V**.
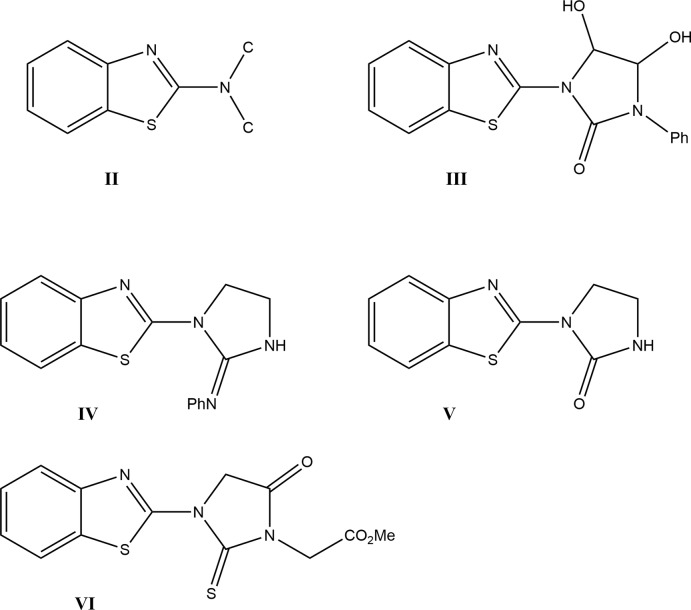


## Synthesis and crystallization   

To a mixture of 2-amino­benzo­thia­zole (2.22 mmol), bis(2-chloro­eth­yl)amine (1.11 mmol) and potassium carbonate (3.21 mmol) in DMF (25 mL) was added a catalytic amount of tetra-*n*-butyl­ammonium bromide (0.37 mmol). The mixture was stirred at 353 K for 24 h. The solid material was removed by filtration and the solvent evaporated *in vacuo*. The solid product was purified by recrystallization from ethanol to give colourless crystals (yield: 70%).

## Refinement   

Crystal data, data collection and structure refinement details are summarized in Table 4 ▸. All hydrogen atoms were located in a difference-Fourier map and their coordinates and isotropic displacement parameters refined without restraints.

## Supplementary Material

Crystal structure: contains datablock(s) I, global. DOI: 10.1107/S2056989020001723/jj2219sup1.cif

Structure factors: contains datablock(s) I. DOI: 10.1107/S2056989020001723/jj2219Isup2.hkl

Supporting information file. DOI: 10.1107/S2056989020001723/jj2219Isup3.cdx

Supporting information file. DOI: 10.1107/S2056989020001723/jj2219Isup4.cml

CCDC reference: 1982595

Additional supporting information:  crystallographic information; 3D view; checkCIF report

## Figures and Tables

**Figure 4:**
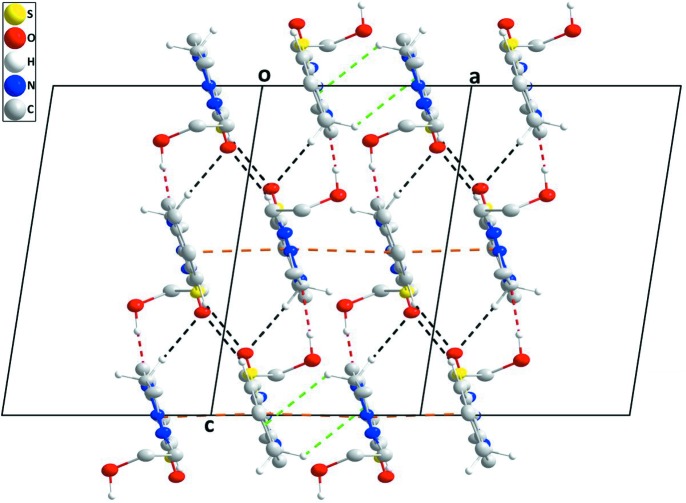
A partial packing diagram viewed along the *b* axis with inter­molecular inter­actions depicted as in Fig. 3 ▸. Three unit cells along the *a* axis are shown.

**Table 1:** Hydrogen-bond geometry (Å, °) *Cg*1 is the centroid of the benzene ring (*A*, C1–C6).

*D*—H⋯*A*	*D*—H	H⋯*A*	*D*⋯*A*	*D*—H⋯*A*
O2—H2*A*⋯N1^iii^	0.90 (2)	1.97 (2)	2.8560 (15)	170 (2)
C5—H5⋯O1^i^	0.954 (19)	2.559 (19)	3.4439 (16)	154.3 (14)
C8—H8*B*⋯O1^vi^	0.958 (17)	2.532 (16)	3.2542 (16)	132.2 (13)
C8—H8*A*⋯*Cg*1^iv^	0.997 (17)	2.840 (16)	3.5646 (15)	130.0 (12)

Symmetry codes: (i) 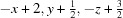; (iii) 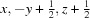; (iv) 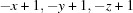; (vi) 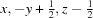.

**Table 2:** Selected interatomic distances (Å)

S1⋯O1	2.7721 (10)	C1⋯C7^iv^	3.4070 (17)
S1⋯C11^i^	3.6700 (14)	C2⋯C10^iv^	3.5859 (18)
S1⋯C1^ii^	3.6552 (12)	C3⋯C10^iv^	3.5928 (19)
S1⋯H11*A* ^i^	3.117 (16)	C4⋯C10^ii^	3.4350 (19)
O1⋯C9^iii^	3.2869 (16)	C4⋯C8^iv^	3.587 (2)
O1⋯C8^iii^	3.2543 (16)	C6⋯C7^ii^	3.5502 (17)
O2⋯N1^iii^	2.8560 (14)	C1⋯H2*A* ^vi^	2.81 (2)
O2⋯C3^iv^	3.4071 (17)	C4⋯H8*A* ^iv^	2.850 (16)
O2⋯N3	2.9500 (15)	C5⋯H8*A* ^iv^	2.718 (15)
O1⋯H9*B* ^iii^	2.640 (17)	C9⋯H12*B*	2.830 (16)
O1⋯H11*A*	2.482 (17)	C12⋯H9*A*	2.857 (18)
O1⋯H8*B* ^iii^	2.534 (16)	H2⋯H9*A* ^vii^	2.49 (3)
O2⋯H9*A* ^v^	2.804 (19)	H2⋯H2*A* ^vi^	2.53 (3)
O2⋯H12*B* ^v^	2.803 (18)	H2*A*⋯H8*B* ^iii^	2.59 (3)
O2⋯H3^iv^	2.601 (18)	H4⋯C12^viii^	2.828 (18)
O2⋯H2^iii^	2.838 (18)	H4⋯H12*A* ^viii^	2.38 (2)
N1⋯C12^vi^	3.4313 (17)	H4⋯H12*B* ^viii^	2.38 (2)
N2⋯C5^ii^	3.4204 (17)	H5⋯O1^i^	2.557 (17)
N1⋯H8*B*	2.769 (17)	H5⋯H11*A* ^i^	2.36 (2)
N1⋯H2*A* ^vi^	1.97 (2)	H8*B*⋯H11*A* ^vi^	2.44 (2)
N1⋯H8*A*	2.857 (17)	H9*A*⋯H12*B*	2.40 (2)

Symmetry codes: (i) 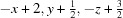; (ii) 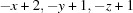; (iii) 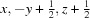; (iv) 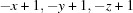; (v) 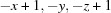; (vi) 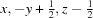; (vii) 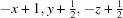; (viii) 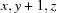.

**Table 3:** Comparison of the selected (X-ray and DFT) geometric data (Å, °)

Bonds/angles	X-ray	B3LYP/6–311G(d,p)
S1—C6	1.7448 (13)	1.83061
S1—C7	1.7517 (12)	1.85613
O1—C10	1.2246 (16)	1.24399
O2—C12	1.4161 (17)	1.45513
N1—C7	1.3060 (16)	1.30197
N1—C1	1.3940 (16)	1.40321
N2—C7	1.3619 (17)	1.37118
N2—C10	1.3930 (16)	1.40686
N2—C8	1.4628 (16)	1.47735
N3—C10	1.3477 (16)	1.37333
N3—C11	1.4485 (16)	1.45760
N3—C9	1.4543 (17)	1.47023
		
C6—S1—C7	88.16 (6)	87.72
C7—N2—C10	125.18 (10)	126.57
C7—N1—C1	109.77 (10)	110.27
C7—N2—C8	122.65 (10)	121.09
C10—N2—C8	112.16 (10)	112.17
C10—N3—C11	123.10 (11)	122.39
C10—N3—C9	113.37 (10)	113.06
C11—N3—C9	122.24 (11)	123.84

**Table 4:** Experimental details

Crystal data	
Chemical formula	C_12_H_13_N_3_O_2_S
*M*_r_	263.31
Crystal system, space group	Monoclinic, *P*2_1_/*c*
Temperature (K)	150
*a*, *b*, *c* (Å)	7.2863 (2), 13.9178 (5), 11.6156 (4)
β (°)	98.866 (1)
*V* (Å^3^)	1163.85 (7)
*Z*	4
Radiation type	Cu *K*α
μ (mm^−1^)	2.47
Crystal size (mm)	0.28 × 0.27 × 0.11

Data collection	
Diffractometer	Bruker D8 VENTURE PHOTON 100 CMOS
Absorption correction	Multi-scan (*SADABS*; Krause *et al.*, 2015 ▸)
*T*_min_, *T*_max_	0.69, 0.77
No. of measured, independent and observed [*I* > 2σ(*I*)] reflections	8781, 2246, 2160
*R*_int_	0.023
(sin θ/λ)_max_ (Å^−1^)	0.618

Refinement	
*R*[*F* ^2^ > 2σ(*F* ^2^)], *wR*(*F* ^2^), *S*	0.030, 0.080, 1.06
No. of reflections	2246
No. of parameters	215
H-atom treatment	All H-atom parameters refined
Δρ_max_, Δρ_min_ (e Å^−3^)	0.20, −0.36

Computer programs: *APEX3* and *SAINT* (Bruker, 2016 ▸), *SHELXT* (Sheldrick, 2015*a* ▸), *SHELXL2018* (Sheldrick, 2015*b* ▸), *DIAMOND* (Brandenburg & Putz, 2012 ▸) and *SHELXTL* (Sheldrick, 2008 ▸).

## supplementary crystallographic information

### Crystal data

**Table:**

C_12_H_13_N_3_O_2_S	*F*(000) = 552
*M**_r_* = 263.31	*D*_x_ = 1.503 Mg m^−^^3^
Monoclinic, *P*2_1_/*c*	Cu *K*α radiation, λ = 1.54178 Å
*a* = 7.2863 (2) Å	Cell parameters from 7989 reflections
*b* = 13.9178 (5) Å	θ = 6.2–72.3°
*c* = 11.6156 (4) Å	µ = 2.47 mm^−^^1^
β = 98.866 (1)°	*T* = 150 K
*V* = 1163.85 (7) Å^3^	Block, colourless
*Z* = 4	0.28 × 0.27 × 0.11 mm

### Data collection

**Table:**

Bruker D8 VENTURE PHOTON 100 CMOS diffractometer	2246 independent reflections
Radiation source: INCOATEC IµS micro-focus source	2160 reflections with *I* > 2σ(*I*)
Mirror monochromator	*R*_int_ = 0.023
Detector resolution: 10.4167 pixels mm^-1^	θ_max_ = 72.3°, θ_min_ = 6.2°
ω scans	*h* = −9→8
Absorption correction: multi-scan (*SADABS*; Krause *et al.*, 2015)	*k* = −17→15
*T*_min_ = 0.69, *T*_max_ = 0.77	*l* = −14→14
8781 measured reflections	

### Refinement

**Table:**

Refinement on *F*^2^	Primary atom site location: dual
Least-squares matrix: full	Secondary atom site location: difference Fourier map
*R*[*F*^2^ > 2σ(*F*^2^)] = 0.030	Hydrogen site location: difference Fourier map
*wR*(*F*^2^) = 0.080	All H-atom parameters refined
*S* = 1.06	*w* = 1/[σ^2^(*F*_o_^2^) + (0.0449*P*)^2^ + 0.4296*P*] where *P* = (*F*_o_^2^ + 2*F*_c_^2^)/3
2246 reflections	(Δ/σ)_max_ = 0.001
215 parameters	Δρ_max_ = 0.20 e Å^−^^3^
0 restraints	Δρ_min_ = −0.36 e Å^−^^3^

### Special details

**Table:**

Geometry. All esds (except the esd in the dihedral angle between two l.s. planes) are estimated using the full covariance matrix. The cell esds are taken into account individually in the estimation of esds in distances, angles and torsion angles; correlations between esds in cell parameters are only used when they are defined by crystal symmetry. An approximate (isotropic) treatment of cell esds is used for estimating esds involving l.s. planes.
Refinement. Refinement of F^2^ against ALL reflections. The weighted R-factor wR and goodness of fit S are based on F^2^, conventional R-factors R are based on F, with F set to zero for negative F^2^. The threshold expression of F^2^ > 2sigma(F^2^) is used only for calculating R-factors(gt) etc. and is not relevant to the choice of reflections for refinement. R-factors based on F^2^ are statistically about twice as large as those based on F, and R- factors based on ALL data will be even larger.

### Fractional atomic coordinates and isotropic or equivalent isotropic displacement parameters (Å^2^)

**Table:**

	*x*	*y*	*z*	*U*_iso_*/*U*_eq_	
S1	0.84367 (4)	0.49137 (2)	0.62655 (3)	0.02128 (12)	
O1	0.88222 (13)	0.29904 (7)	0.68752 (8)	0.0249 (2)	
O2	0.55711 (14)	0.04397 (7)	0.66690 (8)	0.0288 (2)	
H2A	0.586 (3)	0.0432 (16)	0.745 (2)	0.055 (6)*	
N1	0.67379 (15)	0.47968 (7)	0.41147 (9)	0.0191 (2)	
N2	0.74321 (14)	0.33066 (7)	0.49825 (9)	0.0193 (2)	
N3	0.78449 (16)	0.17939 (8)	0.55357 (9)	0.0223 (2)	
C1	0.69815 (16)	0.57713 (9)	0.43706 (11)	0.0188 (3)	
C2	0.63908 (18)	0.65197 (10)	0.35997 (12)	0.0241 (3)	
H2	0.570 (2)	0.6355 (13)	0.2837 (16)	0.035 (4)*	
C3	0.67664 (19)	0.74589 (10)	0.39635 (13)	0.0267 (3)	
H3	0.637 (2)	0.7985 (14)	0.3464 (16)	0.035 (5)*	
C4	0.77217 (19)	0.76532 (10)	0.50716 (13)	0.0275 (3)	
H4	0.797 (2)	0.8334 (13)	0.5348 (16)	0.035 (4)*	
C5	0.82913 (19)	0.69236 (10)	0.58568 (12)	0.0251 (3)	
H5	0.891 (2)	0.7065 (13)	0.6624 (16)	0.032 (4)*	
C6	0.79036 (17)	0.59783 (9)	0.54928 (11)	0.0201 (3)	
C7	0.74425 (16)	0.42847 (9)	0.50177 (10)	0.0177 (3)	
C8	0.66402 (19)	0.27650 (9)	0.39448 (11)	0.0217 (3)	
H8A	0.528 (2)	0.2896 (12)	0.3761 (15)	0.031 (4)*	
H8B	0.725 (2)	0.2958 (12)	0.3310 (15)	0.027 (4)*	
C9	0.7093 (2)	0.17217 (10)	0.43041 (11)	0.0274 (3)	
H9A	0.597 (3)	0.1309 (13)	0.4205 (16)	0.037 (5)*	
H9B	0.801 (2)	0.1427 (13)	0.3901 (15)	0.036 (4)*	
C10	0.81190 (17)	0.27069 (9)	0.59095 (11)	0.0189 (3)	
C11	0.8591 (2)	0.09704 (10)	0.62142 (11)	0.0250 (3)	
H11A	0.900 (2)	0.1210 (12)	0.6983 (15)	0.028 (4)*	
H11B	0.972 (2)	0.0720 (13)	0.5935 (15)	0.031 (4)*	
C12	0.7190 (2)	0.01623 (9)	0.62181 (12)	0.0256 (3)	
H12A	0.784 (2)	−0.0373 (12)	0.6672 (14)	0.026 (4)*	
H12B	0.681 (2)	−0.0069 (11)	0.5418 (16)	0.025 (4)*	

### Atomic displacement parameters (Å^2^)

**Table:**

	*U*^11^	*U*^22^	*U*^33^	*U*^12^	*U*^13^	*U*^23^
S1	0.0263 (2)	0.01777 (18)	0.01832 (18)	0.00109 (11)	−0.00106 (13)	−0.00110 (10)
O1	0.0344 (5)	0.0211 (5)	0.0181 (4)	0.0011 (4)	0.0003 (4)	0.0002 (3)
O2	0.0353 (5)	0.0272 (5)	0.0228 (5)	−0.0006 (4)	0.0011 (4)	0.0037 (4)
N1	0.0222 (5)	0.0164 (5)	0.0185 (5)	0.0007 (4)	0.0025 (4)	0.0010 (4)
N2	0.0259 (5)	0.0152 (5)	0.0163 (5)	−0.0001 (4)	0.0012 (4)	0.0004 (4)
N3	0.0319 (6)	0.0153 (5)	0.0183 (5)	−0.0003 (4)	−0.0002 (4)	0.0023 (4)
C1	0.0191 (6)	0.0160 (6)	0.0218 (6)	0.0007 (5)	0.0048 (4)	−0.0003 (5)
C2	0.0284 (7)	0.0198 (6)	0.0237 (6)	0.0030 (5)	0.0027 (5)	0.0023 (5)
C3	0.0312 (7)	0.0181 (6)	0.0316 (7)	0.0048 (5)	0.0073 (5)	0.0036 (5)
C4	0.0300 (7)	0.0174 (6)	0.0361 (7)	0.0016 (5)	0.0081 (6)	−0.0030 (5)
C5	0.0262 (6)	0.0204 (6)	0.0281 (7)	0.0004 (5)	0.0027 (5)	−0.0051 (5)
C6	0.0198 (6)	0.0183 (6)	0.0221 (6)	0.0017 (5)	0.0030 (5)	−0.0006 (5)
C7	0.0180 (6)	0.0166 (6)	0.0188 (6)	−0.0002 (4)	0.0041 (4)	−0.0005 (4)
C8	0.0299 (7)	0.0175 (6)	0.0168 (6)	−0.0019 (5)	0.0005 (5)	−0.0003 (5)
C9	0.0439 (8)	0.0179 (6)	0.0187 (6)	−0.0025 (6)	−0.0008 (5)	0.0003 (5)
C10	0.0220 (6)	0.0172 (6)	0.0180 (6)	0.0006 (5)	0.0046 (5)	0.0021 (5)
C11	0.0333 (7)	0.0167 (6)	0.0243 (7)	0.0038 (5)	0.0022 (5)	0.0033 (5)
C12	0.0399 (8)	0.0153 (6)	0.0210 (7)	0.0011 (5)	0.0023 (6)	0.0003 (5)

### Geometric parameters (Å, º)

**Table:**

S1—C6	1.7448 (13)	C3—C4	1.392 (2)
S1—C7	1.7517 (12)	C3—H3	0.952 (19)
O1—C10	1.2246 (16)	C4—C5	1.385 (2)
O2—C12	1.4161 (17)	C4—H4	1.008 (18)
O2—H2A	0.90 (2)	C5—C6	1.3974 (18)
N1—C7	1.3060 (16)	C5—H5	0.954 (19)
N1—C1	1.3940 (16)	C8—C9	1.5327 (18)
N2—C7	1.3619 (17)	C8—H8A	0.997 (17)
N2—C10	1.3930 (16)	C8—H8B	0.958 (17)
N2—C8	1.4628 (16)	C9—H9A	0.993 (19)
N3—C10	1.3477 (16)	C9—H9B	0.967 (18)
N3—C11	1.4485 (16)	C11—C12	1.5194 (19)
N3—C9	1.4543 (17)	C11—H11A	0.957 (18)
C1—C2	1.3975 (18)	C11—H11B	0.992 (17)
C1—C6	1.4013 (18)	C12—H12A	0.991 (17)
C2—C3	1.388 (2)	C12—H12B	0.982 (18)
C2—H2	0.977 (19)		
			
S1···O1	2.7721 (10)	C1···C7^iv^	3.4070 (17)
S1···C11^i^	3.6700 (14)	C2···C10^iv^	3.5859 (18)
S1···C1^ii^	3.6552 (12)	C3···C10^iv^	3.5928 (19)
S1···H11A^i^	3.117 (16)	C4···C10^ii^	3.4350 (19)
O1···C9^iii^	3.2869 (16)	C4···C8^iv^	3.587 (2)
O1···C8^iii^	3.2543 (16)	C6···C7^ii^	3.5502 (17)
O2···N1^iii^	2.8560 (14)	C1···H2A^vi^	2.81 (2)
O2···C3^iv^	3.4071 (17)	C4···H8A^iv^	2.850 (16)
O2···N3	2.9500 (15)	C5···H8A^iv^	2.718 (15)
O1···H9B^iii^	2.640 (17)	C9···H12B	2.830 (16)
O1···H11A	2.482 (17)	C12···H9A	2.857 (18)
O1···H8B^iii^	2.534 (16)	H2···H9A^vii^	2.49 (3)
O2···H9A^v^	2.804 (19)	H2···H2A^vi^	2.53 (3)
O2···H12B^v^	2.803 (18)	H2A···H8B^iii^	2.59 (3)
O2···H3^iv^	2.601 (18)	H4···C12^viii^	2.828 (18)
O2···H2^iii^	2.838 (18)	H4···H12A^viii^	2.38 (2)
N1···C12^vi^	3.4313 (17)	H4···H12B^viii^	2.38 (2)
N2···C5^ii^	3.4204 (17)	H5···O1^i^	2.557 (17)
N1···H8B	2.769 (17)	H5···H11A^i^	2.36 (2)
N1···H2A^vi^	1.97 (2)	H8B···H11A^vi^	2.44 (2)
N1···H8A	2.857 (17)	H9A···H12B	2.40 (2)
			
C6—S1—C7	88.16 (6)	N2—C7—S1	121.60 (9)
C12—O2—H2A	106.8 (13)	N2—C8—C9	102.87 (10)
C7—N1—C1	109.77 (10)	N2—C8—H8A	109.6 (10)
C7—N2—C10	125.18 (10)	C9—C8—H8A	113.3 (10)
C7—N2—C8	122.65 (10)	N2—C8—H8B	108.6 (10)
C10—N2—C8	112.16 (10)	C9—C8—H8B	111.6 (10)
C10—N3—C11	123.10 (11)	H8A—C8—H8B	110.5 (14)
C10—N3—C9	113.37 (10)	N3—C9—C8	103.60 (10)
C11—N3—C9	122.24 (11)	N3—C9—H9A	109.4 (11)
N1—C1—C2	124.89 (12)	C8—C9—H9A	112.1 (10)
N1—C1—C6	115.18 (11)	N3—C9—H9B	108.8 (10)
C2—C1—C6	119.93 (12)	C8—C9—H9B	114.0 (11)
C3—C2—C1	118.69 (13)	H9A—C9—H9B	108.7 (15)
C3—C2—H2	123.1 (10)	O1—C10—N3	128.27 (12)
C1—C2—H2	118.2 (11)	O1—C10—N2	124.38 (11)
C2—C3—C4	120.76 (13)	N3—C10—N2	107.35 (10)
C2—C3—H3	120.8 (11)	N3—C11—C12	113.03 (11)
C4—C3—H3	118.5 (11)	N3—C11—H11A	105.6 (10)
C5—C4—C3	121.51 (13)	C12—C11—H11A	111.7 (10)
C5—C4—H4	117.3 (10)	N3—C11—H11B	111.1 (10)
C3—C4—H4	121.1 (10)	C12—C11—H11B	109.4 (10)
C4—C5—C6	117.70 (13)	H11A—C11—H11B	105.7 (14)
C4—C5—H5	120.9 (11)	O2—C12—C11	113.43 (11)
C6—C5—H5	121.4 (11)	O2—C12—H12A	111.6 (9)
C5—C6—C1	121.38 (12)	C11—C12—H12A	106.8 (9)
C5—C6—S1	128.70 (10)	O2—C12—H12B	108.1 (10)
C1—C6—S1	109.92 (9)	C11—C12—H12B	109.3 (9)
N1—C7—N2	121.45 (11)	H12A—C12—H12B	107.4 (13)
N1—C7—S1	116.94 (10)		
			
C7—N1—C1—C2	−179.95 (12)	C8—N2—C7—S1	−178.90 (9)
C7—N1—C1—C6	0.42 (15)	C6—S1—C7—N1	−1.36 (10)
N1—C1—C2—C3	−178.49 (12)	C6—S1—C7—N2	177.54 (10)
C6—C1—C2—C3	1.12 (19)	C7—N2—C8—C9	175.82 (11)
C1—C2—C3—C4	0.4 (2)	C10—N2—C8—C9	−5.38 (14)
C2—C3—C4—C5	−1.5 (2)	C10—N3—C9—C8	−7.90 (16)
C3—C4—C5—C6	1.1 (2)	C11—N3—C9—C8	−175.30 (11)
C4—C5—C6—C1	0.40 (19)	N2—C8—C9—N3	7.49 (14)
C4—C5—C6—S1	179.83 (10)	C11—N3—C10—O1	−8.4 (2)
N1—C1—C6—C5	178.12 (11)	C9—N3—C10—O1	−175.68 (13)
C2—C1—C6—C5	−1.53 (19)	C11—N3—C10—N2	172.01 (11)
N1—C1—C6—S1	−1.40 (13)	C9—N3—C10—N2	4.74 (15)
C2—C1—C6—S1	178.95 (9)	C7—N2—C10—O1	−0.1 (2)
C7—S1—C6—C5	−178.02 (13)	C8—N2—C10—O1	−178.84 (12)
C7—S1—C6—C1	1.45 (9)	C7—N2—C10—N3	179.53 (11)
C1—N1—C7—N2	−178.10 (11)	C8—N2—C10—N3	0.77 (14)
C1—N1—C7—S1	0.80 (13)	C10—N3—C11—C12	135.47 (13)
C10—N2—C7—N1	−178.69 (11)	C9—N3—C11—C12	−58.36 (17)
C8—N2—C7—N1	−0.05 (18)	N3—C11—C12—O2	−59.05 (15)
C10—N2—C7—S1	2.46 (17)		

Symmetry codes: (i) −*x*+2, *y*+1/2, −*z*+3/2; (ii) −*x*+2, −*y*+1, −*z*+1; (iii) *x*, −*y*+1/2, *z*+1/2; (iv) −*x*+1, −*y*+1, −*z*+1; (v) −*x*+1, −*y*, −*z*+1; (vi) *x*, −*y*+1/2, *z*−1/2; (vii) −*x*+1, *y*+1/2, −*z*+1/2; (viii) *x*, *y*+1, *z*.

### Hydrogen-bond geometry (Å, º)

*Cg*1 is the centroid of the benzene ring (*A*, C1–C6).

**Table:**

*D*—H···*A*	*D*—H	H···*A*	*D*···*A*	*D*—H···*A*
O2—H2*A*···N1^iii^	0.90 (2)	1.97 (2)	2.8560 (15)	170 (2)
C5—H5···O1^i^	0.954 (19)	2.559 (19)	3.4439 (16)	154.3 (14)
C8—H8*B*···O1^vi^	0.958 (17)	2.532 (16)	3.2542 (16)	132.2 (13)
C8—H8*A*···*Cg*1^iv^	0.997 (17)	2.840 (16)	3.5646 (15)	130.0 (12)

Symmetry codes: (i) −*x*+2, *y*+1/2, −*z*+3/2; (iii) *x*, −*y*+1/2, *z*+1/2; (iv) −*x*+1, −*y*+1, −*z*+1; (vi) *x*, −*y*+1/2, *z*−1/2.

## References

[bb1] Ayhan-Kilcigil, G., Kus, C., Çoban, T., Can-Eke, B. & Iscan, M. (2004). *J. Enzyme Inhib. Med. Chem.* **19**, 129–135. 10.1080/1475636042000202017 15449727

[bb2] Becke, A. D. (1993). *J. Chem. Phys.* **98**, 5648–5652.

[bb3] Bénéteau, V., Besson, T., Guillard, J., Léonce, S. & Pfeiffer, B. (1999). *Eur. J. Med. Chem.* **34**, 1053–1060.

[bb4] Brandenburg, K. & Putz, H. (2012). *DIAMOND*, Crystal Impact GbR, Bonn, Germany.

[bb5] Bruker (2016). *APEX3*, *SAINT* and *SADABS*. Bruker AXS, Inc., Madison, Wisconsin, USA.

[bb6] Ćaleta, I., Grdiša, M., Mrvoš-Sermek, D., Cetina, M., Tralić-Kulenović, V., Pavelić, K. & Karminski-Zamola, G. (2004). *Farmaco*, **59**, 297–305. 10.1016/j.farmac.2004.01.008 15081347

[bb7] Chakib, I., El Bakri, Y., Lai, C.-H., Benbacer, L., Zerzouf, A., Essassi, E. M. & Mague, J. T. (2019). *J. Mol. Struct.* **1198**, 126910–126921.

[bb8] Chakib, I., Zerzouf, A., Zouihri, H., Essassi, E. M. & Ng, S. W. (2010*a*). *Acta Cryst.* E**66**, o2842. 10.1107/S1600536810040316 PMC3008974 21589028

[bb9] Chakibe, I., Zerzouf, A., Essassi, E. M., Reichelt, M. & Reuter, H. (2010b). *Acta Cryst.* E**66**, o1096. 10.1107/S1600536810013176 PMC2979087 21579149

[bb53] Delmas, F., Avellaneda, A., Di Giorgio, C., Robin, M., De Clercq, E., Timon-David, P. & Galy, J. P. (2004). *Eur. J. Med. Chem.* **39**, 685–690. 10.1016/j.ejmech.2004.04.006 15276301

[bb10] Desai, N. C., Bhavsar, A. M. & Baldaniya, B. B. (2009). *Indian J. Pharm. Sci.* **71**, 90–94. 10.4103/0250-474X.51953 PMC2810063 20177470

[bb11] Ellouz, M., Sebbar, N. K., Boulhaoua, M., Essassi, E. M. & Mague, J. T. (2017). *IUCrData*, **2**, x170646.

[bb12] Frisch, M. J., Trucks, G. W., Schlegel, H. B., Scuseria, G. E., Robb, M. A., Cheeseman, J. R., *et al.* (2009). *GAUSSIAN-09*. Gaussian Inc., Wallingford, CT, US.

[bb13] Groom, C. R., Bruno, I. J., Lightfoot, M. P. & Ward, S. C. (2016). *Acta Cryst.* B**72**, 171–179. 10.1107/S2052520616003954 PMC4822653 27048719

[bb14] Harfenist, M., Soroko, E. F. & McKenzie, G. M. (1978). *J. Med. Chem.* **21**, 405–409. 10.1021/jm00202a021 650671

[bb15] Hari Narayana Moorthy, N. S., Saxena, V., Karthikeyan, C. & Trivedi, P. (2012). *J. Enzyme Inhib. Med. Chem.* **27**, 201–207. 10.3109/14756366.2011.584191 21635210

[bb16] Hathwar, V. R., Sist, M., Jørgensen, M. R. V., Mamakhel, A. H., Wang, X., Hoffmann, C. M., Sugimoto, K., Overgaard, J. & Iversen, B. B. (2015). *IUCrJ*, **2**, 563–574. 10.1107/S2052252515012130 PMC4547824 26306198

[bb17] Hirshfeld, H. L. (1977). *Theor. Chim. Acta*, **44**, 129–138.

[bb18] Hni, B., Sebbar, N. K., Hökelek, T., El Ghayati, L., Bouzian, Y., Mague, J. T. & Essassi, E. M. (2019). *Acta Cryst.* E**75**, 593–599. 10.1107/S2056989019004250 PMC6505589 31110793

[bb19] Huang, S.-T., Hsei, I.-J. & Chen, C. (2006). *Bioorg. Med. Chem.* **14**, 6106–6119. 10.1016/j.bmc.2006.05.007 16714116

[bb20] Jayatilaka, D., Grimwood, D. J., Lee, A., Lemay, A., Russel, A. J., Taylor, C., Wolff, S. K., Cassam-Chenai, P. & Whitton, A. (2005). *TONTO - A System for Computational Chemistry. Available at: http://hirshfeldsurface.net/*

[bb21] Kaur, H., Kumar, S., Singh, I., Saxena, K. K. & Kumar, A. (2010). *DIG. J. Nanomater. Bios* **5**, 67–76.

[bb22] Kok, S. H. L., Gambari, R., Chui, C. H., Yuen, M. C. W., Lin, E., Wong, R. S. M., Lau, F. Y., Cheng, G. Y. M., Lam, W. S., Chan, S. H., Lam, K. H., Cheng, C. H., Lai, P. B. S., Yu, W. Y., Cheung, F., Tang, J. C. O. & Chan, A. S. C. (2008). *Bioorg. & Med. Chem.* **16**, 3626–3631. 10.1016/j.bmc.2008.02.005 18295491

[bb23] Kozísek, J., Ulický, L., Floch, L. & Langer, V. (1995). *Acta Cryst.* C**51**, 1429–1431.

[bb24] Krause, L., Herbst-Irmer, R., Sheldrick, G. M. & Stalke, D. (2015). *J. Appl. Cryst.* **48**, 3–10. 10.1107/S1600576714022985 PMC4453166 26089746

[bb25] Latrofa, A., Franco, M., Lopedota, A., Rosato, A., Carone, D. & Vitali, C. (2005). *Farmaco*, **60**, 291–297. 10.1016/j.farmac.2005.01.010 15848203

[bb26] Mackenzie, C. F., Spackman, P. R., Jayatilaka, D. & Spackman, M. A. (2017). *IUCrJ*, **4**, 575–587. 10.1107/S205225251700848X PMC5600021 28932404

[bb27] McKinnon, J. J., Jayatilaka, D. & Spackman, M. A. (2007). *Chem. Commun.* pp. 3814–3816. 10.1039/b704980c 18217656

[bb28] Mekhzoum, M. E. M., El Bourakadi, K., Essassi, E. M., Qaiss, A. E. K. & Bouhfid, R. (2019). *J. Mol. Struct.* **1193**, 303–309.

[bb29] Mekhzoum, M. E. M., Essassi, E. M., Qaiss, A. E. K. & Bouhfid, R. (2016). *RSC Adv.* **6**, 111472–111481.

[bb30] Nagarajan, S. R., De Crescenzo, G. A., Getman, D. P., Lu, H. F., Sikorski, J. A., Walker, J. L., McDonald, J. J., Houseman, K. A., Kocan, G. P., Kishore, N., Mehta, P. P., Funkes-Shippy, C. L. & Blystone, L. (2003). *Bioorg. & Med. Chem.* **11**, 4769–4777. 10.1016/j.bmc.2003.07.001 14556792

[bb31] Naithani, D. K., Shrivastava, V. K., Barthwal, J. P., Szxena, A. K., Gupta, T. A. & Shanker, K. (1989). *Indian J. Chem.* **28B**, 990–992.

[bb32] Oketani, K., Nagakura, N., Harada, K. & Inoue, T. (2001). *Eur. J. Pharmacol.* **422**, 209–216. 10.1016/s0014-2999(01)01022-6 11430933

[bb33] Olyaei, A., Abbasi, A., Ghandi, M., Salimi, F. & Eriksson, L. (2006). *Acta Cryst.* E**62**, o5326–o5327.

[bb34] Pitta, E., Geronikaki, A., Surmava, S., Eleftheriou, P., Mehta, V. P. & Van der Eycken, E. V. (2013). *J. Enzyme Inhib. Med. Chem.* **28**, 113–122. 10.3109/14756366.2011.636362 22380777

[bb35] Saczewski, F., Kornicka, A. & Gdaniec, M. (2005). *Pol. J. Chem.* **79**, 115–120.

[bb36] Saggu, J. S., Sharma, R., Dureja, H. & Kumar, V. (2002). *J. Indian Inst. Sci.* **82**, 177–182.

[bb37] Sahoo, S. K., Jena, H. S., Majji, G. & Patel, B. K. (2014). *Synthesis*, **46**, 1886–1900.

[bb38] Sebbar, N. K., Ellouz, M., Elmsellem, H., Zerzouf, A., Hlimi, F. & Essassi, E. M. (2018). *J. Mar. Chim. Heterocycl.* **17**, 179–183.

[bb39] Sebbar, N. K., Mekhzoum, M. E. M., Essassi, E. M., Zerzouf, A., Talbaoui, A., Bakri, Y., Saadi, M. & Ammari, L. E. (2016). *Res. Chem. Intermed.* **42**, 6845–6862.

[bb40] Shastry, C. S., Joshi, S. D., Aravind, M. B. & Veerapur, V. P. (2003). *Indian. J. Het. Chem.* **13**, 57–60.

[bb41] Sheldrick, G. M. (2008). *Acta Cryst.* A**64**, 112–122. 10.1107/S0108767307043930 18156677

[bb42] Sheldrick, G. M. (2015*a*). *Acta Cryst.* A**71**, 3–8.

[bb43] Sheldrick, G. M. (2015*b*). *Acta Cryst.* C**71**, 3–8.

[bb44] Singh, M. K., Tilak, R., Nath, G., Awasthi, S. K. & Agarwal, A. (2013). *Eur. J. Med. Chem.* **63**, 635–644. 10.1016/j.ejmech.2013.02.027 23567952

[bb45] Spackman, M. A. & Jayatilaka, D. (2009). *CrystEngComm*, **11**, 19–32.

[bb46] Spackman, M. A., McKinnon, J. J. & Jayatilaka, D. (2008). *CrystEngComm*, **10**, 377–388.

[bb47] Tewari, A. K. & Mishra, A. (2006). *Indian J. Chem.* **45B**, 489–493.

[bb48] Turner, M. J., Grabowsky, S., Jayatilaka, D. & Spackman, M. A. (2014). *J. Phys. Chem. Lett.* **5**, 4249–4255. 10.1021/jz502271c 26273970

[bb49] Turner, M. J., McKinnon, J. J., Wolff, S. K., Grimwood, D. J., Spackman, P. R., Jayatilaka, D. & Spackman, M. A. (2017). *CrystalExplorer17*. The University of Western Australia.

[bb50] Turner, M. J., Thomas, S. P., Shi, M. W., Jayatilaka, D. & Spackman, M. A. (2015). *Chem. Commun.* **51**, 3735–3738. 10.1039/c4cc09074h 25525647

[bb51] Venkatesan, P., Thamotharan, S., Ilangovan, A., Liang, H. & Sundius, T. (2016). *Spectrochim. Acta Part A*, **153**, 625–636. 10.1016/j.saa.2015.09.002 26452098

[bb52] Yang, B. Q., Yang, P. H. & Zhu, A. L. (2003). *Chin. Chem. Lett.* **14**, 901–903.

